# Novel Approaches to Postnatal Prophylaxis to Eliminate Vertical Transmission of HIV

**DOI:** 10.9745/GHSP-D-22-00401

**Published:** 2023-04-28

**Authors:** Theodore Ruel, Martina Penazzato, Jennifer M. Zech, Moherndran Archary, Tim R. Cressey, Ameena Goga, Joseph Harwell, Raphael J. Landovitz, Maria Grazia Lain, Marc Lallemant, Eleanor Namusoke-Magongo, Irene Mukui, Sallie R. Permar, Andrew J. Prendergast, Roger Shapiro, Elaine J. Abrams

**Affiliations:** a University of California, San Francisco, San Francisco, CA, USA.; b World Health Organization, Geneva, Switzerland.; c ICAP at Columbia University, Mailman School of Public Health, Columbia University, NY, USA.; d University of KwaZulu-Natal, Durban, South Africa.; e AMS-IRD Research Collaboration, Faculty of Associated Medical Sciences, Chiang Mai University, Chiang Mai, Thailand.; f HIV and other Infectious Diseases Research Unit, South African Medical Research Council, Cape Town, South Africa.; g Department of Paediatrics and Child Health, University of Pretoria, South Africa.; h Clinton Health Access Initiative, Boston, MA, USA.; i UCLA Center for Clinical AIDS Research and Education, David Geffen School of Medicine at UCLA, Los Angeles, CA, USA.; j Fundação Ariel Glaser contra o SIDA Pediátrico, Maputo, Mozambique.; k AMS-PHPT Research Collaboration, Chiang Mai University, Chiang Mia, Thailand.; l Penta Foundation Italy, Padova, Italy.; m Paediatric and Adolescent HIV Care Treatment Branch, Ministry of Health, Kampala, Uganda.; n Drugs for Neglected Diseases Initiative, Nairobi, Kenya.; o Department of Pediatrics, Weill Cornell Medicine, New York, NY, USA.; p Queen Mary University of London, London, United Kingdom.; q Zvitambo Institute for Maternal and Child Health Research, Harare, Zimbabwe.; r Department of Immunology and Infectious Diseases, Harvard T.H. Chan School of Public Health, Boston, MA, USA.; s Vagelos College of Physicians and Surgeons, Columbia University, New York, NY, USA.

## Abstract

Despite progress in providing antiretroviral therapy to pregnant women living with HIV, a substantial number of vertical transmissions continue to occur. Novel approaches leveraging modern potent, safe, and well-tolerated antiretroviral drugs are urgently needed.

## Key Messages

- Postnatal prophylaxis continues to play an essential role in efforts to eliminate new pediatric HIV infections and maximize HIV-free survival.
- New approaches to postnatal prophylaxis using currently available modern oral antiretroviral drugs could surmount many current barriers.
- Long-acting agents and delivery platforms currently in development for treating people living with HIV have great potential as postnatal prophylaxis for infants.
- Studies of novel postnatal prophylactic agents are possible and imperative in the near future and must be prioritized early in the antiretroviral drug development pipeline.
- Close collaboration between researchers, community representatives, industry, regulators, and policymakers will be the critical ingredient to ensure HIV-free survival for all infants with HIV exposure.

## INTRODUCTION

With new advances in antiretroviral drugs for HIV prevention and treatment, as well as increasing coverage globally, the landscape around vertical transmission of HIV is rapidly changing. It is estimated that 82% of pregnant women globally received antiretroviral therapy (ART) in 2021.^1^ Increasingly, women in sub-Saharan Africa are transitioning to optimized dolutegravir-based antiretroviral regimens, offering greater potency and durability. Women with HIV also have access to better care, including integrated antenatal and HIV services, viral load monitoring, multi-month ART dispensing, and patient-centered differentiated service delivery models with postnatal follow-up of the mother-infant dyad.^2^ However, a substantial number of vertical transmissions continue to occur perinatally and during breastfeeding, driven by shortfalls in testing, treatment coverage, adherence, and retention in care among mothers. Among an estimated 160,000 children with new HIV infections globally in 2021, 48% had mothers who had not started ART, 22% had mothers who discontinued their treatment, and 8% had mothers on treatment but were not able to maintain virologic suppression.^1^ Furthermore, incident HIV infections among pregnant and breastfeeding women contribute to an increasing proportion of new pediatric infections.^3^

**FIGURE 1:**
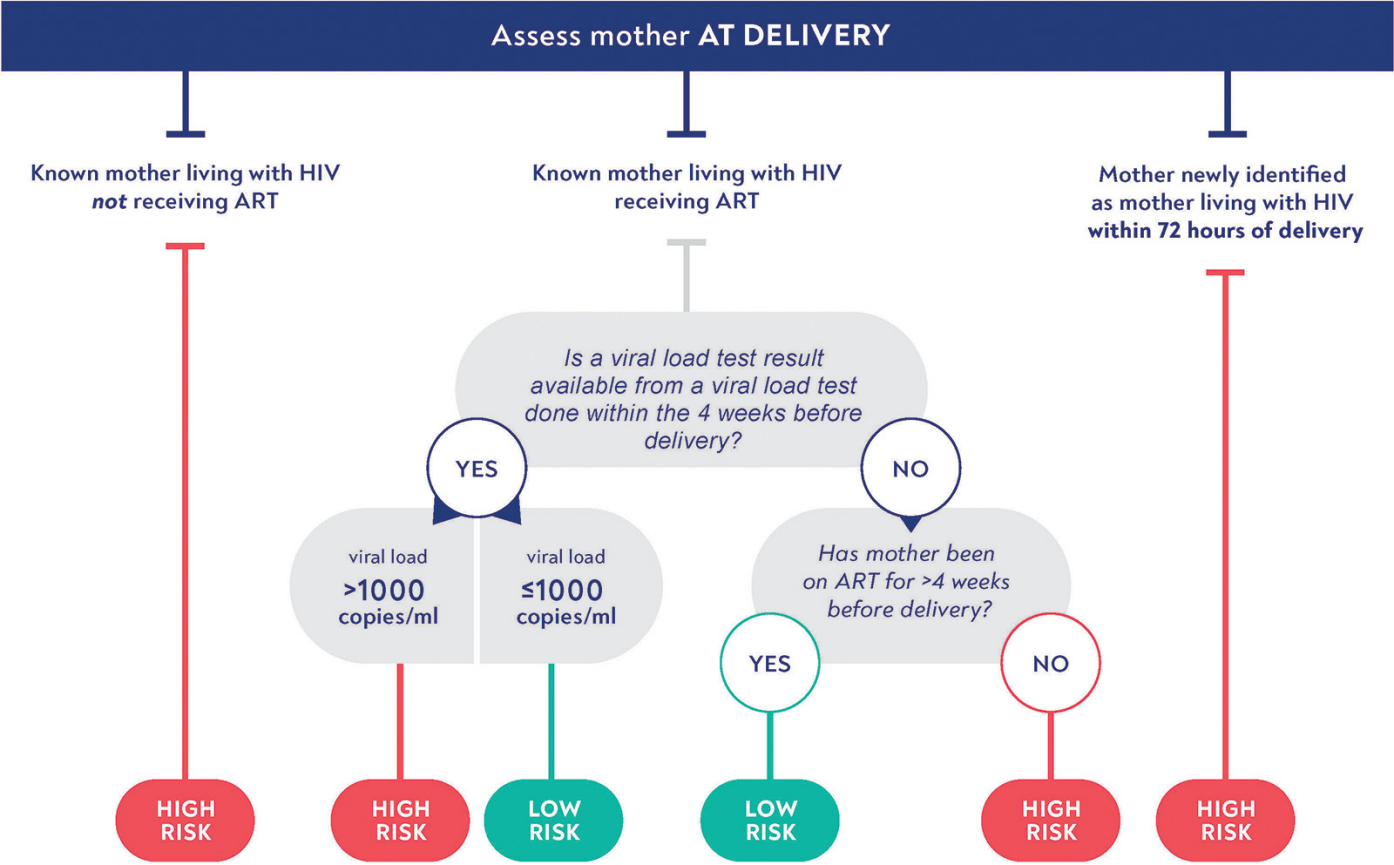
Current Algorithm for HIV Transmission Risk Stratification^a^ ^a^Adapted from: World Health Organization’s *Consolidated Guidelines on HIV Prevention, Testing, Treatment, Service Delivery and Monitoring: Recommendations for a Public Health Approach.*^5^

Postnatal prophylaxis—the provision of antiretroviral drugs to HIV-exposed infants—remains a key tool to reduce vertical transmission. In the absence of effective maternal ART, postnatal prophylaxis has been demonstrated effective in the prevention of vertical transmission around the time of delivery and during breastfeeding. Current World Health Organization (WHO) guidelines for postnatal prophylaxis are designed primarily to reduce the risk of transmission around the time of delivery, with regimens that are risk-stratified depending on maternal treatment status, timing of ART initiation, and virological suppression (Figure 1).^4^

**FIGURE 2:**
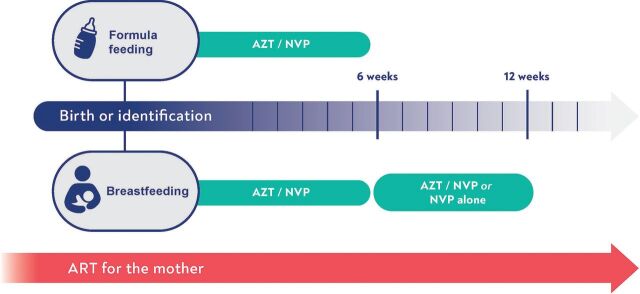
Current Postnatal Prophylaxis Guidance for Infants at High Risk of HIV Transmission^a^ Abbreviations: AZT: zidovudine; NVP: nevirapine; ART: antiretroviral treatment. ^a^ Figure design based on guidance from: World Health Organization’s *Consolidated Guidelines on HIV Prevention, Testing, Treatment, Service Delivery and Monitoring: Recommendations for a Public Health Approach*.^5^

Infants born to women who start ART late in pregnancy or at the time of delivery are at high risk of HIV acquisition. For these high-risk situations, the WHO guidelines recommend giving postnatal prophylaxis to the infant for 12 weeks (Figure 2).^5^

These guidelines have been adopted widely, with countries customizing to their contexts. But their effective implementation has been limited for several reasons. Risk stratification, which identifies infants requiring more complex postnatal prophylactic regimens at birth, can be difficult to implement, especially in resource-limited settings with limited access to viral load monitoring. Furthermore, there are challenges to administering antiretroviral drugs to newborns. Those currently in use, nevirapine and zidovudine, require daily or twice daily oral administration and are delivered as syrups dispensed in bulky bottles with the potential for unintentional disclosure of the maternal HIV status. Because these drugs are no longer considered optimal for treating HIV infection, the decreasing global market for them has led to challenges in supply security. Finally, postnatal prophylaxis is not currently routinely recommended by the WHO for infants aged older than 12 weeks. This leaves breastfeeding infants vulnerable to transmission if their mothers experience viremia secondary to lapses in adherence or treatment failure or if there are drug stock-outs. Some countries have adapted the guidelines to include prophylaxis during breastfeeding.

To further reduce the risk of HIV acquisition during breastfeeding, there is a need for more effective approaches to infant prophylaxis that are easier to implement, safe when used for prolonged durations, take advantage of modern drugs, and capitalize on novel delivery platforms. In 2021, the WHO and the International Maternal Pediatric Adolescent AIDS Clinical Trials Network convened a workshop to accelerate the research and development of new agents and new approaches to postnatal prophylaxis. The second meeting of the series was held June 8–10, 2021. In this article, we present the data reviewed and the ideas generated by workshop participants to identify potential innovative strategies for postnatal prophylaxis to prevent HIV vertical transmission.

More effective approaches to postnatal HIV prophylaxis are needed that are easier to implement, safe for prolonged use, take advantage of modern drugs, and capitalize on novel delivery platforms.

## WHAT IS THE EVIDENCE BASE FOR CURRENT APPROACHES TO POSTNATAL PROPHYLAXIS?

The first evidence that antiretroviral drugs reduced the risk of vertical transmission—and one of the earliest success stories in the HIV epidemic response—came from the Pediatric AIDS Clinical Trials Group 076 study.^6^ Zidovudine was given to pregnant women from 14 weeks of gestation through labor and delivery and to their infants for the first 6 weeks after birth; this strategy reduced transmission risk by more than two-thirds. Subsequent trials demonstrated benefit from shorter courses of maternal/infant antiretroviral drugs,^6^^–^^8^ from the use of infant prophylaxis alone,^9^ and from early initiation of infant prophylaxis after birth.^10^ Studies in the late 1990s and early 2000s demonstrated the benefit of single-dose nevirapine prophylaxis but also identified the first concerns about the development of HIV drug resistance among infants acquiring HIV infection despite postnatal prophylaxis.^11^ The benefit of combination prophylaxis was shown with both daily zidovudine following single-dose nevirapine and daily zidovudine plus daily lamivudine.^12^^–^^16^ In 2010, the HIV Prevention Trials Network 040/Pediatric AIDS Clinical Trials Group 1043 study demonstrated that for infants of mothers who delivered before starting treatment, 2-drug or 3-drug short-course regimens to the infant provided equivalent protection against infection, with peripartum transmission rates of ∼2%–3%.^17^ Several studies around this time turned their attention to extended prophylaxis during the breastfeeding period to infants of mothers not receiving ART, demonstrating breastfeeding transmission rates that ranged from 0.5% to 5.2% with daily infant nevirapine given for 6 or 14 weeks or throughout breastfeeding.^18^^–^^21^ Similar results have been reported for both daily lamivudine as well as lopinavir/ritonavir used for prophylaxis during breastfeeding.^22^ Of note, no additional benefit was reported when zidovudine was added to nevirapine for infants during breastfeeding in 1 trial.^18^ In the era of universal ART and with increasingly effective antiretroviral regimens for pregnant and breastfeeding women, the low rate of vertical transmission has made it more difficult to conduct studies to evaluate new agents and approaches to infant prophylaxis. Novel study approaches, such as conducting a Bayesian trial design used to analyze data from the PHPT5 study in Thailand, are being considered to allow new strategies for postnatal prophylaxis to be studied efficiently.^23^^,^^24^

## WHAT ARE THE CHALLENGES SPECIFIC TO STUDYING PHARMACOKINETICS AND DETERMINING DOSING FOR NEONATES?

Many currently approved antiretroviral drugs like dolutegravir used in the treatment of children with HIV have untested potential as potent agents for postnatal prophylaxis. A new generation of long-acting antiretroviral drugs, such as injectable cabotegravir, has been approved by the U.S. Food and Drug Administration for treatment and prophylaxis for adolescents and adults. Recent studies suggest that combinations of broadly neutralizing antibodies could potentially be effective for prophylaxis in adults. But the pharmacokinetics of all of these products must be established in neonates and infants before they can be trialed for efficacy. Drug development and dose-finding for neonates is particularly challenging not only because of difficulties in performing clinical research in this vulnerable population but also because of highly variable pharmacokinetics during the first months of life. Additionally, the necessity for multiple blood draws, safety procedures, and frequent study visits soon after birth can be a burden for families. Drug dosing for children older than 2 years can be relatively accurately estimated by extrapolating adult doses using allometric scaling according to body weight, which empirically describes the nonlinear relationship between drug elimination and body size. However, this is not applicable for neonates as the rapid maturation of the physiological processes underlying drug absorption, distribution, metabolism, and excretion immediately after birth must also be considered. Rapid increases in antiretroviral drug metabolism and/or elimination can occur during the first few weeks of life, necessitating very low doses at birth followed by frequent dose increases during the first weeks of life.^25^^,^^26^ The challenges are even greater for preterm neonates, in whom the maturation timelines of gestational and postnatal age overlap.^27^ Thus, the pharmacokinetics of antiretroviral drugs cannot be simply extrapolated from studies in older children, and the optimal dose in neonates must be confirmed through dedicated clinical trials.^28^

Drug development and dose-finding for neonates is particularly challenging because of difficulties in performing clinical research in this population and highly variable pharmacokinetics during the first months of life.

Studies of neonatal “washout” are an efficient way to gain insight into the neonatal metabolism of drugs before formal neonatal pharmacokinetic dosing trials. Washout studies assess a drug taken by the mother that crosses the placenta; through repeated infant blood sampling after birth, this washout data can help provide an estimate of drug clearance over the first days and weeks of life. For newer antiretroviral drugs, drug exposure targets for postnatal prophylaxis are similar to those for treatment.^28^ Several ongoing studies aim to expand antiretroviral drug options for neonates. The PETITE study has recently assessed the pediatric solid “4-in-1” granule formulations of abacavir, lamivudine, and ritonavir-boosted-lopinavir (Cipla, Ltd) in term neonates exposed to HIV.^29^ Unfortunately, early data on the 4-in-1 formulation revealed low lopinavir plasma concentrations,^29^ and administering higher doses of the fixed-dose combination is not possible without risk of overexposure to the abacavir and lamivudine components. Subsequently, the PETITE study is now assessing the separate solid formation of abacavir/lamivudine pediatric dispersible tablets and lopinavir/ritonavir granules in neonates. Dolutegravir-based treatment is currently recommended for the treatment of children aged at least 4 weeks and weighing at least 3.0 kg; studies of the pharmacokinetics and safety of dolutegravir are planned. The long dosing intervals offered by new long-acting antiretroviral drugs and broadly neutralizing antibodies would address many barriers in the implementation of neonatal prophylaxis, but these products will also require pharmacokinetic study in neonates and infants. For example, data about the drug release following intramuscular injections of long-acting drugs in neonates is needed to ensure therapeutic levels are rapidly achieved after birth. Initial pharmacokinetic and safety studies of subcutaneous injections of VRC01 and VRC01LS in infants are encouraging. Pharmacokinetic characteristics suggest the agents could be dosed infrequently (e.g., every 12 weeks) during breastfeeding.^30^^,^^31^

## WHAT CAN WE LEARN FROM STUDIES OF HIV ANTIRETROVIRAL PROPHYLAXIS IN ADULTS ABOUT TARGET PRODUCT PROFILES?

Within the context of HIV prevention in adults, the ideal product profile for an agent includes efficacy, safety, high partitioning into genital tissue compartments, prolonged activity with convenient dosing, high barrier to resistance, unique resistance profile (i.e., one that would not compromise other drugs, especially first-line regimens), no significant drug-drug interactions with commonly coadministered medications, and low cost.^32^ The product must also be easy to implement, encompassing issues of acceptability, discretion, low likelihood of perpetuating stigma, and congruence with sexual practices. Tenofovir-based preexposure prophylaxis possesses many of these attributes but lacks discretion (oral administration is visible) and has suboptimal acceptability secondary to the requirement for daily dosing. These deficiencies, despite extraordinary efficacy, have limited its population-level benefit. The dapivirine ring has demonstrated a modest 30% reduction in HIV incidence, has high acceptability in some cisgender female populations, and is now recommended by the WHO.^33^^,^^34^ Long-acting injectable cabotegravir has been shown to be superior to daily oral tenofovir-based preexposure prophylaxis and has recently obtained U.S. Food and Drug Administration approval. However, ensuring adherence to injectable cabotegravir is also critical, and rare prophylaxis failures may compromise the activity of first-line treatment regimens in the same drug class.^35^^–^^37^ With a long half-life, islatravir promised the possibility of monthly oral or every 6–12 month implants; unfortunately, signs of lymphocyte toxicity placed the development of this agent on hold.^38^ A recent decision was made to continue the development of islatravir for treatment, but the prevention program was discontinued.^39^ Subcutaneous lenacapavir dosed every 6 months is in advanced-stage clinical trials. One concerning feature of all long-acting formulations is the “tail” of subtherapeutic concentrations that occur when subsequent doses are missed. This tail of low concentrations could not only lower efficacy as prophylaxis but also select for resistance in the case of HIV-acquisition during this period. For these reasons, clinical outcomes in the context of missed doses must be evaluated for all long-acting products. The role of broadly neutralizing antibodies in adult preexposure prophylaxis remains unclear, given the successes of small-molecule agents.^40^ It is yet untested whether a combination of multiple antibodies can effectively prevent HIV transmission in human populations.

## WHAT IS THE POTENTIAL ROLE OF PASSIVE IMMUNIZATION IN POSTNATAL PROPHYLAXIS?

While current approaches to prophylaxis against HIV infection in adults and children are based primarily on small-molecule drugs, it is important to remember that antibodies can provide powerful protection against viral infections. The gold standard for both prevention of vertical transmission and the generation of life-long immunity is the hepatitis B prevention program, which is highly effective when implemented during prenatal, delivery, and neonatal care visits. This maternal-infant targeted program employs both risk-based and universal prevention strategies: (1) passive immunization with hyperimmune globulin at delivery in infants of mothers with evidence of active hepatitis B infection; (2) antiviral treatment of mothers with high viral replication; and (3) universal active immunization at delivery and throughout infancy that can generate life-long protective immunity.^41^ Theoretically, this framework could be applied to the prevention of perinatal/postnatal HIV transmission. The addition of passive immunization of the infant with a combination of broadly neutralizing antibodies at birth to maternal and infant antiretroviral drugs is an attractive option, particularly in high-risk situations, such as detectable maternal viral load, acute maternal infection, or in areas of high HIV prevalence where acute infections are more likely.^42^^,^^43^ Safety and pharmacokinetic studies in infants demonstrated that the subcutaneous administration of a CD4 binding site-directed antibody, VRC01 and its long-acting version VRCO1LS, in the first few days after birth had good tolerability, was safe, and persisted above a protective level for 8 weeks in more than 95% of infants.^44^ Moreover, studies in a nonhuman primate model of infant HIV infection revealed that broadly neutralizing antibody-based interventions could both provide prophylaxis and act as treatment, most effectively when administered within hours after birth.^45^ Of note, a broad array of broadly neutralizing antibodies is in the pipeline, and discussion is ongoing about how best to test different combinations of products for preventing postnatal infection.^46^ Finally, as active immunization strategies for HIV improve in their ability to generate broadly neutralizing antibody responses and with the understanding that the infant immune system generates broadly neutralizing responses more frequently during infection than that of adults, initiation of a multidose immunization in the vaccine schedule for children aged younger than 5 years may be the ideal population for active vaccination to achieve broad neutralization responses before adolescence.^47^ The combination approach of ART during pregnancy and breastfeeding, short-term antiretroviral prophylaxis to the infant around delivery, paired with broadly neutralizing antibody-based passive immunization for high-risk infants, and universal active HIV vaccination for all infants—mirroring hepatitis B prevention programs—could be the formula to eliminate pediatric HIV infections and generate long-term immunity.

The addition of passive immunization of the infant with a combination of broadly neutralizing antibodies at birth to maternal and infant antiretroviral drugs is an attractive option particularly in high-risk situations.

## DO POSTNATAL PROPHYLAXIS STRATEGIES NEED TO BE RISK STRATIFIED?

One of the central challenges in designing new approaches to postnatal prophylaxis is how to accommodate infants at different levels of risk during each period of exposure—in utero, intrapartum, and during breastfeeding. Traditionally, guidelines have recommended different regimens for the immediate postnatal period based on maternal risk factors (documented viremia and/or the recent start of ART). Furthermore, there is little evidence and hence limited guidance to address new or escalating risk if a mother becomes viremic during breastfeeding. A “1 regimen for all” approach would be ideal to overcome the challenges of implementing a risk-stratified approach to postnatal prophylaxis, depending on the feasibility and safety of the regimen. One strategy would be to use potent 3-drug antiretroviral regimens that could serve dual roles as “presumptive treatment” for infants with in-utero HIV infection while providing a high level of protection for those at high risk of intrapartum/early postnatal acquisition. However, using 3-drug antiretroviral regimens as prophylaxis for infants at low risk would be resource intensive and places those infants at potential additional risk of toxicity. It is possible that some agents in the pipeline, such as broadly neutralizing antibodies, could be both highly potent and safe enough to be used as routine postnatal prophylaxis for all infants at birth and throughout breastfeeding, independent of the risk of HIV acquisition. Arguments in favor of and against maintaining risk-based approaches to postnatal prophylaxis are summarized in Table 1.

**TABLE 1.** Rationale for and Against Using a Risk-Based Postnatal Prophylaxis Algorithm

**In Favor**	**Against**
Risk of HIV acquisition is not uniform among exposed infants; different approaches are needed to address different scenarios; patient-centered approach tailoring response to individual infant.	Risk may become more uniform (and low) in near future, with rapid scale-up of more potent, efficacious, and tolerable maternal treatment with dolutegravir.
Low-risk infants avoid unnecessary antiretroviral drug exposure and the associated potential toxicities.	A low rate of transmission persists even among low-risk infants, suggesting potential benefit from additional agents to all exposed infants.
High-risk infants benefit from more aggressive management with multiple drugs/agents.	No evidence exists to support the efficacy of multiple drug perinatal prophylaxis when mothers are on effective treatment, with studies performed in the era of dolutegravir-based treatment.
Stratification aligns risk (toxicity): benefit (prophylaxis efficacy) of approaches with the transmission risk.	Risk is difficult to assess and dynamic. Perinatal risk assessment depends on testing and medical records that are not always available. Over the duration of breastfeeding, individual maternal risk can change and can be difficult to assess without frequent visits and viral load testing.
Stratified approaches optimize health system resource use, aligning cost of more intensive regimens with target population that will derive the most benefit.	Risk assessment adds complexity and is itself resource intensive, requiring testing and visits for mothers. It can be challenging for health systems and clinics to stock and implement multiple regimens for infants.

A “1 regimen for all” approach would be ideal to overcome the challenges of implementing a risk-stratified approach to postnatal prophylaxis but would depend on the feasibility and safety of the regimen.

## SHOULD ALL INFANTS RECEIVE POSTNATAL PROPHYLAXIS WHILE BREASTFEEDING?

There are also many questions about the added value of providing prophylaxis to infants during breastfeeding. While current WHO guidelines recommend breastfeeding without any prophylaxis, after completion of the perinatal regimen, if a mother is on ART and virally suppressed, sustaining complete adherence throughout breastfeeding can be difficult, and episodes of viremia are not uncommon.^5^^,^^48^^,^^49^ Prophylaxis to the infant could be protective if maternal viral suppression is not sustained, but, to date, there is no evidence of the added value of infant prophylaxis when the mother is on treatment, nor is it clear how high adherence would be to this strategy when mothers are poorly adherent to their own regimen. Particularly with increasing interest in breastfeeding in high-income settings, the role of infant prophylaxis during breastfeeding is being questioned. In many settings, infants are being prescribed prolonged antiretroviral drug regimens during breastfeeding using a risk-based (when maternal viremia is detected) or a “treat all approach” of prophylaxis to all breastfeeding infants throughout the period of exposure. Arguments for and against infant prophylaxis during breastfeeding are summarized in Table 2.

**TABLE 2.** Rationale for and Against Whether All Infants Should Receive Postnatal Prophylaxis While Breastfeeding

**In Favor**	**Against**
A large portion (∼50%) of vertical transmission currently occurs during the breastfeeding period.	Studies have not shown that adding infant prophylaxis to effective maternal treatment further reduces transmission risk.
Risk of transmission throughout breastfeeding is dynamic, with maternal viremia difficult to monitor or predict; maternal viremia during breastfeeding is common even among mothers who maintain suppression during pregnancy.	More effective oral treatment with dolutegravir and new long-acting formulations offer the prospect of unprecedented coverage and durability of virologic suppression in breastfeeding women.
Maternal adherence to treatment is difficult to sustain throughout the breastfeeding period; approaches to support nonadherent women to achieve viral suppression and to predict lapses in adherence are inadequate.	Predictors of maternal nonadherence have been identified (including younger age, new HIV diagnosis, late presentation to care, and non-disclosure) and can be used to target additional prevention measures.
Infants deserve resources and interventions that offer direct protection and do not rely on maternal treatment.	Limited resources should focus on optimizing maternal adherence and access to good care.
Routine infant care can serve as a platform to maintain infants on prophylaxis throughout breastfeeding.	It is difficult to maintain infant prophylaxis over long periods of time; there is significant loss to follow-up by 1 year of life.
New injectable and long-acting formulations limit the visibility of infants receiving prophylaxis and could reduce concerns about stigma.	Providing prophylaxis to infants raises issues of disclosure of maternal infection status.
Many mothers fall out of care, thus, interventions that do not depend on maternal clinic attendance are needed.	New point-of-care viral load testing will make monitoring of mothers easier.
Simplified, safer options that have potential for greater efficacy for postnatal prophylaxis are in development.	Addressing underlying drivers of maternal treatment failure will benefit both the infant and the mother.

## WHAT ARE POTENTIAL APPROACHES TO POSTNATAL PROPHYLAXIS IN THE NEAR AND FAR FUTURE?

**FIGURE 3:**
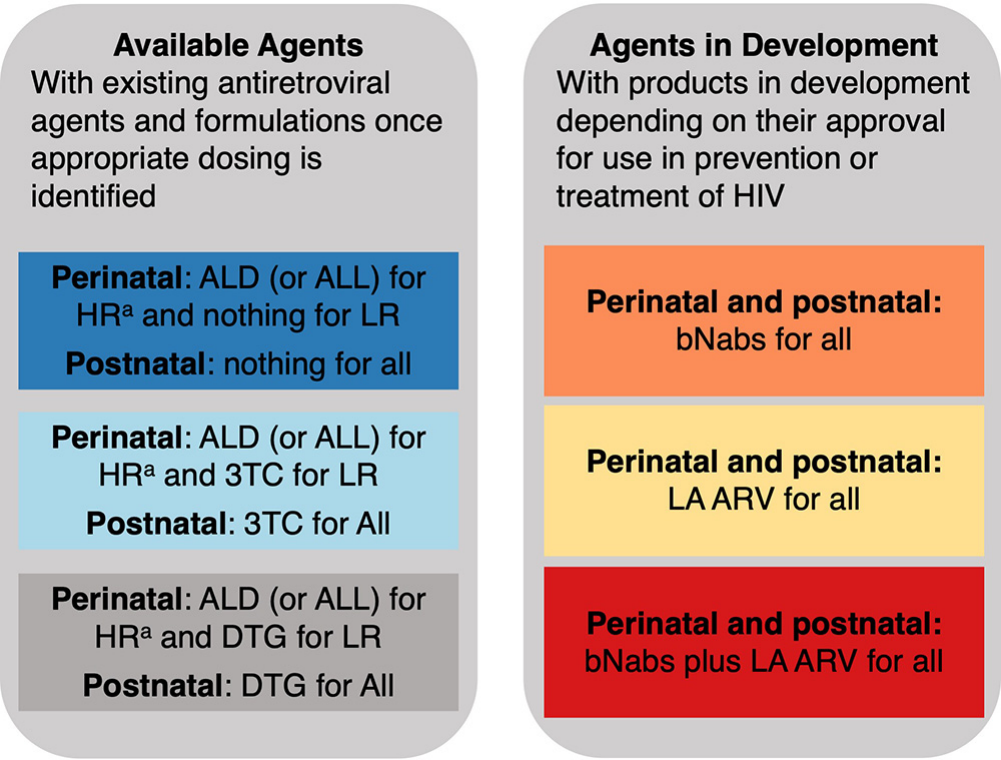
Novel Approaches to Infant Postnatal Prophylaxis Abbreviations: ALD, abacavir plus lamivudine plus dolutegravir tablets; ALL, abacavir plus lamivudine plus lopinavir-ritonavir granules; bNabs, broadly neutralizing antibodies; LA ARV, long-acting antiretroviral drugs; LR, low-risk situations; HR, high-risk situations. ^a^ For premature babies, zidovudine and nevirapine should be used.

There are several antiretroviral drugs currently available for the treatment of infants with HIV and others in the pipeline that hold potential for postnatal prophylaxis.^50^ We summarize these agents in Table 3 and depict potential strategies for employing them in Figure 3.

**TABLE 3.** Agents With Potential Novel Roles as Postnatal Prophylaxis

**Agent**	**Potential Route and Frequency of Delivery**	**Advantages**	**Disadvantages**
Available now			
Dolutegravir	Oral, daily	Dispersible 5 mg tablet; broadly available with good supply chain; high resistance barrier; low toxicity.	No evidence of efficacy for prevention. Dosing for infants aged younger than 4 weeks is not yet available; infections could lead to selection of drug resistance, jeopardizing use as treatment.
Lamivudine	Oral, twice daily	Available in syrup and solid formulations; dosing available down from 32 weeks gestation; broadly available with good supply chain; low toxicity; demonstrated efficacy for postnatal prophylaxis.	Low barrier to resistance.
Lopinavir/ritonavir	Oral, twice daily	Available in liquid formulation and granules; demonstrated efficacy as postnatal prophylaxis.	Twice daily dosing is less convenient. Persistent concerns about toxicity in neonates aged younger than 14 days and premature, possibly related to vehicle of liquid formulation.
Abacavir	Oral, once daily	Well-tolerated in children, with fewer long-term side effects compared to zidovudine.	No evidence of efficacy as prophylaxis; evidence of long-term toxicity in adults.
In development pipeline			
Broadly neutralizing antibodies	Intramuscular, every 1–2 months (maybe longer)	Dosing for VRCO1, VRCO1LS, and VRC07-523LS is available; new class with high barriers to resistance and separate pathway from current treatments; easy to dose across growth periods; well tolerated; no drug-drug interactions. Appeal of injections to some settings: doesn’t rely on adherence to daily oral, fewer adherence/stigma issues compared to oral medications kept at home.	Current options must be combined to optimize antiviral effects; efficacy for prevention indication still needs to be demonstrated; requires cold chain and site capacity to deliver. Biosimilar production will be a significant challenge.
Islatravir	Oral, monthly	Could be delivered in clinics, facilitating dose adjustments, or at home. New class with high barriers to resistance and separate pathway from current treatments. Appeal of injections to some settings: doesn’t rely on adherence to daily oral, fewer adherence/stigma issues compared to oral medications kept at home.	Drug development halted due to evidence of lymphocyte toxicity (ongoing investigation may allow continuation of the drug development program in the future).
Lenacapavir	Subcutaneously, every 3–6 months	As a new drug class, lower risk for interaction with maternal or infant treatment; surmounts issues of adherence to oral regimens.	May need more frequent dosing during first year of life to accommodate growth.
Cabotegravir	Intramuscular, once every 1–2 months	Appeal of injections to some settings: doesn’t rely on adherence to daily oral, fewer adherence/stigma issues compared to oral medications kept at home.	Pharmacokinetics will be challenging, especially for infants aged younger than 4 weeks and premature infants. Infections could lead to selection of resistance, compromising other agents like dolutegravir. Generic production will be a challenge in the immediate future.

### Currently Available Options

Dolutegravir is a highly potent antiretroviral drug that may play an important role in postnatal prevention.^51^ While dosing in neonates is under study, the dolutegravir once-daily dispersible tablet is easy to administer and well tolerated in children aged 4 weeks and older.^52^ It could be used as single-drug prophylaxis in the perinatal period for infants at low risk of perinatal transmission and throughout breastfeeding, but such a strategy would require assessing the risk of selecting for dolutegravir resistance in the event of breakthrough infections. Lamivudine dosing, safety, and efficacy in neonates and infants have been well established, and it has proven efficacy for postnatal prevention.^53^^–^^55^ It could be paired with dolutegravir for a higher degree of antiviral activity and potentially protect against selection of resistance mutations in those who acquire HIV. Abacavir is another agent that has a long record for treatment and safety in children; recent data support dosing in full-term neonates aged younger than 4 weeks.^56^ The combination of lamivudine and abacavir with dolutegravir could be used perinatally for high-risk situations. This combination is already used for treating children globally, increasing provider comfort, and easing supply chain issues. Dolutegravir alone or paired with lamivudine could be used for prolonged postnatal prophylaxis during breastfeeding. Lopinavir/ritonavir has well-established efficacy for both treatment and prevention of HIV during infancy^54^^,^^57^ and could also be utilized in combination regimens as presumptive treatment/postnatal prophylaxis in the perinatal period for high-risk situations.^58^ Given the challenging pharmacokinetics of lopinavir/ritonavir and dolutegravir in premature infants, it is likely that nevirapine, lamivudine, and zidovudine will remain the best current option for premature neonates until pharmacokinetics and safety of newer drugs are defined for that population. Furthermore, all of the currently available options require daily oral administration throughout the period of exposure posing substantial adherence challenges as well as the potential for inadvertent disclosure of maternal HIV status.

Nevirapine, lamivudine, and zidovudine will likely remain the best current options for premature neonates until pharmacokinetics and safety of newer drugs are defined for that population.

### Options in Development

In the development pipeline for treating HIV, there are several long-acting agents and formulations that are very appealing as prophylaxis for infants. Agents that are injectable, either as intramuscular (e.g., cabotegravir) or subcutaneous (e.g., lenacapavir [GS6207]) formulations, could surmount challenges of adherence that arise from administering daily oral medications to infants. However, it is likely that dosing intervals obtained in adults will be difficult to achieve in the neonatal period, given the changes in clearance and growth that occur over the first weeks and months of life. Given their distinct clearance mechanisms and safety, broadly neutralizing antibodies could be ideal and potentially cost effective if effective combination regimens were to be identified that could be used universally or in high-risk settings.^59^ In future studies, all of these novel agents and approaches would need to be compared to the standard of care and the approach of no postnatal prophylaxis at all for infants at low risk of transmission.

## CONCLUSION

Until all the “leaks” in coverage and treatment failures among mothers with HIV are addressed, postnatal prophylaxis will continue to play an essential role in global efforts to eliminate new pediatric HIV infections and maximize HIV-free survival. Several postnatal prophylaxis strategies have the potential to provide more effective and feasible options for infants and their mothers. In the short term, evaluating universal and risk-stratified approaches that combine legacy and novel antiretroviral drugs that are also used for treatment and passive immunization approaches appears to be the most feasible and expeditious route to advance beyond legacy postnatal prophylaxis regimens. These new strategies will require rapid investigation of the most appropriate dosing in preterm and term neonates with pharmacokinetic modeling and studies. In the long term, pipeline products that allow for less frequent administration open the door to a more integrated approach where a single well-tolerated and effective strategy could be administered across the risk spectrum perinatally and postnatally. These options will require building on the evidence generated in the adult population, defining clearly preferred product characteristics, and actively investigating novel molecules in neonates and infants.^32^ Close collaboration between researchers, community representatives, industry, regulators, and policymakers will be the critical ingredient to ensure HIV-free survival for all infants with HIV exposure.

## Notes

**Peer Reviewed**

**First published online:** April 3, 2023.
